# Supplementary figures and images for: Extracellular matrix and vascular dynamics in the kidney of a murine model for Marfan syndrome
Source: PLoS One. 2023 May 9;18(5):e0285418. doi: 10.1371/journal.pone.0285418 (PMC10168582; doi:10.1371/journal.pone.0285418)

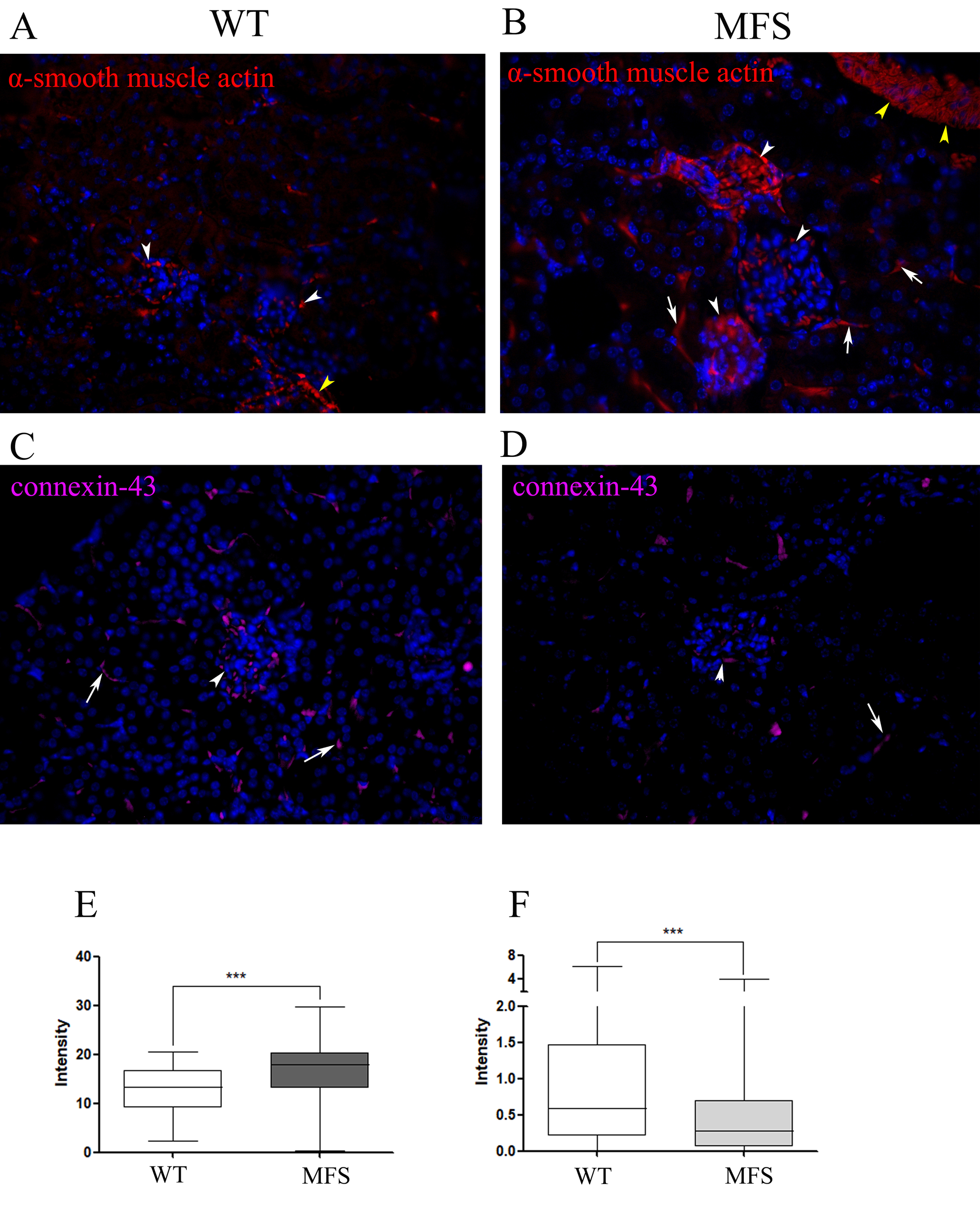

Supplement: S1 Fig — When compared to the WT group (A), the MFS group (B) showed increased staining for α-smooth muscle actin (red) in the glomerulus (white arrowhead), arteriole wall (yellow arrowhead), and myofibroblast cells (white arrows), Connexin-43 (purple) showed a wide distribution in the glomerulus (white arrowheads) and in cells in the parenchyma (white arrows) in the (C) WT group and in the (D) MFS group. Quantification of the intensity of the fluorescence of (E) α-smooth muscle actin and (F) connexin-43. The MFS group revealed a significant increase of α-smooth muscle actin and a significant reduction of Connexin-43. (***) p <0.001. 5 WT and 5 MFS animals were used for Immunofluorescence analysis. The statistical analysis was performed by the Mann-Whitney test. (TIF) [file pone.0285418.s002.tif]

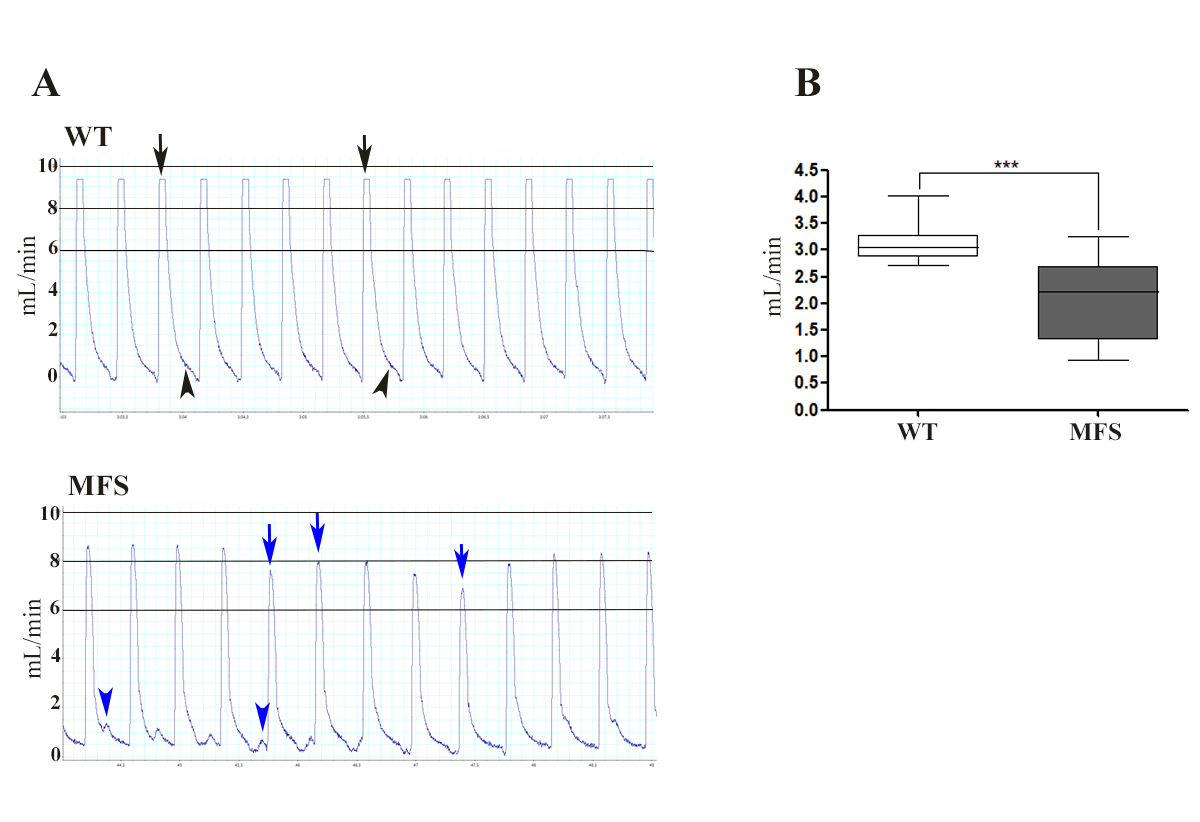

Supplement: S2 Fig — A. Spectral curve analysis of aortic blood flow, in the WT group, presents a uniform monophasic flow pattern with similar peak systolic velocities (PSVs) (black arrows) and no alterations in relaxation curves (black arrowhead). In MFS- we observe a PSV reduced (blue arrow), and alteration in the diastole curve (blue arrowhead). B. Quantification of the aorta blood flow revealed a significantly decreased in the MFS group when compared to the WT group. (*** ρ<0.0001) 10 WT and 10 MFS animals were used for hemodynamic study. The statistical analysis was performed by the Mann-Whitney test. (TIF) [file pone.0285418.s003.tif]

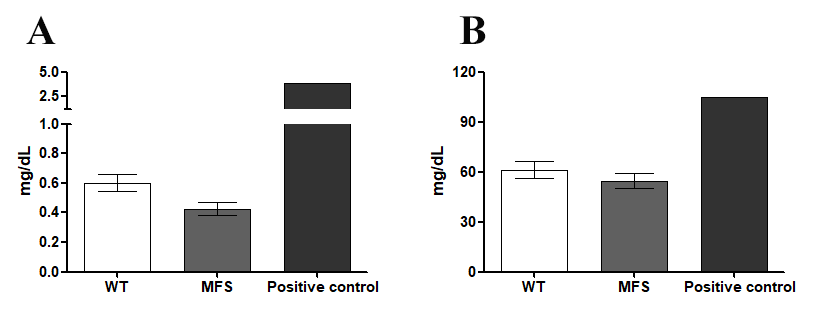

Supplement: S3 Fig — A. serum creatinine of the WT, MFS groups, and positive control. We did not observe a significant difference between the WT and MFS groups. B. serum urea of the WT, MFS groups, and positive control. There was no significant difference between WT and MFS groups. 5 WT and 4 MFS animals were used for the kidney-function study. The statistical analysis was performed by the Mann-Whitney test (A and B). (TIF) [file pone.0285418.s004.tif]

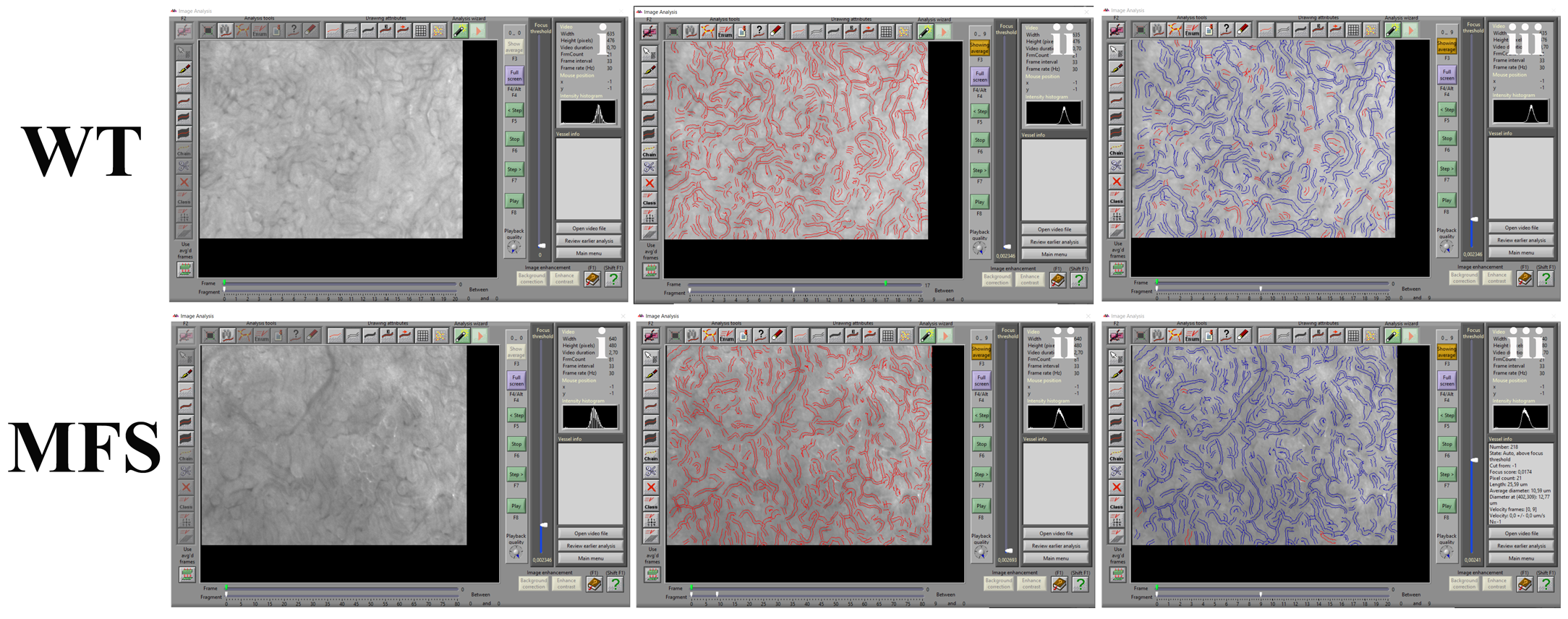

Supplement: S4 Fig — Steps of the processing SDF videos in Automated Vascular Analysis software (AVA) in both WT and MFS groups; i. video attached in AVA; ii. “Enumerate Space-time Diagram” tool in AVA, which identified microvessel; iii. After the “enumerate space-time diagram” tool the finalization of the analysis of the "Total Vessel Density" and "Quantitative Velocity Assessment. (TIF) [file pone.0285418.s005.tif]
