# Supplementary material for: Extracellular matrix and vascular dynamics in the kidney of a murine model for Marfan syndrome
Source: PLoS One. 2023 May 9;18(5):e0285418. doi: 10.1371/journal.pone.0285418 (PMC10168582; doi:10.1371/journal.pone.0285418)
Supplement: S1 File — (PDF) [file pone.0285418.s010.pdf]

Extracellular Matrix and Vascular Dynamics in the Kidney of a murine model for Marfan syndrome

Rodrigo Barbosa de Souza[1¶]; Renan Barbosa Lemes[1]; Orestes Foresto-Neto[3]; Luara Lucena Cassiano[4]; Dieter P Reinhardt [5]; Keith M. Meek[2]; Ivan Hong Jun Koh[6]; Philip N. Lewis*[2] ; Lygia V. Pereira*[1].

Participant consent.

This study did not use patients or retrospective medical records. For that reason, we did not have participant consent.
