# Supplementary material for: Extracellular matrix and vascular dynamics in the kidney of a murine model for Marfan syndrome
Source: PLoS One. 2023 May 9;18(5):e0285418. doi: 10.1371/journal.pone.0285418 (PMC10168582; doi:10.1371/journal.pone.0285418)
Supplement: Attachment — Submitted filename: Response to Reviewers.docx [file pone.0285418.s011.docx]

**Extracellular Matrix and Vascular Dynamics in the Kidney of a murine model for Marfan syndrome**

Rodrigo Barbosa de Souza^1^; Renan Barbosa Lemes^1^; Orestes Foresto-Neto^3^; Luara Lucena Cassiano^4^; Dieter P Reinhardt ^5^; Keith M. Meek^2^; Ivan Hong Jun Koh^6^; Philip N. Lewis*^2^; Lygia V Pereira^1^.

5. Review Comments to the Author

Please use the space provided to explain your answers to the questions above. You may also include additional comments for the author, including concerns about dual publication, research ethics, or publication ethics. (Please upload your review as an attachment if it exceeds 20,000 characters)

We appreciate the Reviewers' efforts in providing a detailed analysis of our paper and thank them for their constructive ideas for its development. To accommodate all of their recommendations, we made significant adjustments to the text, which we discuss in full below. We hope that our paper is now of sufficient high quality to be published as a PLoSOne.

Reviewer #1: The aim of this study is the characterization of the renal phenotype in a relatively new Marfan mouse model (delta lpn) to assess whether the kidney is directly or indirectly is involved or not in the cardiovascular pathogenesis in MFS patients.

The article is of interest for readers involved in the field as well as it is well performed and relatively well explained. In this respect, **the Introduction requires more work explaining more the model** and other studies **involving renal dysfunctions in other mouse models** (i.e. **PMID: 30359839**) and it is not **necessary that explains in the introduction the main obtained findings.**

There is two missing points that should be addressed in some extent in the manuscript, which is mostly simply descriptive (which is fine). **Is there some inflammatory response in affected glomeruli? How is the blood pressure in these Marfan mice?** **Are the angiotensin II blood levels altered? It would be greatly interesting to include some of this data in the paper**.

One point that is not well explained is **when authors affirm that fragmentation of elastic fibers in the aorta is related to an increase in collagen type I…(Discussion section). In fact, usually (not always) collagen expression is increased to compensate in some extent the loss of elasticity**

**Please, next time, put the page number.**

We are most gratified by the Reviewer’s view about our paper “*The article is of interest for readers involved in the field as well as it is well performed and relatively well explained”.*

The Reviewer makes 3 comments relating to adding new text in the introduction.

We appreciate the feedback and have added more details about our model (lines 130–136). Next, in lines 116–122, we comment on the renal impairment that has been seen in other experimental models. In addition, the introduction's data findings were removed.

**Lines 130 to 136.** Mouse models of MFS provide a useful means of investigating the role of Fbn1 mutations in kidney dysfunction. The mg∆^lpn^ is a variant of the mg∆ mouse model. In this model, 6 kb of the fibrillin-1 gene, encompassing exons 19-24, were replaced with a neomycin resistance (neoR) expression cassette (Pereira et al., 1997) flanked by lox-P sequences (Lima et al., 2010). The mg∆^lpn^ is a dominant-negative MFS model that exhibits classic MFS-phenotypes in heterozygosity with alterations in the skeletal, ocular, and cardiovascular systems (Lima et al., 2010; de Souza et al., 2019; Souza et al., 2021a; Souza et al., 2021b). In particular, mg∆^lpn^ mice develop aortic aneurysms and dissection, similar to those seen in patients with a severe MFS phenotype (de Souza et al., 2019).

**Lines 116 to 122.** … Although alterations in other tissues/organs might be expected based on this gene defect, these have been less exploited in the clinical setting [3]. Dysfunctions of the kidney, together with changes to the kidney's fibrillin-rich components by mutations to the *FBN1* gene, have been reported in MFS as well as in several types of glomerular diseases [4,5,6], hydronephrosis [7,8], and renal cysts [3,9]. Other studies with MFS mouse models showed hypoplasia glomerular in a hypomorphic mouse model (mgR/mgR) and cystic kidney disease in the C1039G mouse model (Hartner et al., 2004; Hibender et al., 2019).

In addition, the Reviewer makes 3 questions about the paper.

**Is there some inflammatory response in affected glomeruli?**

In our study, we found no polymorphonuclear or mononuclear inflammatory infiltrates in the renal parenchyma.

The fibrillin-1 mutation has been linked to Marfan syndrome and has the ability to affect TGF-β signaling, promoting its activation (Neptune et al., 2004; Milewicz et al., 2021). TGF-β is a cytokine that, in addition to stimulating macrophages, has been linked to an anti-inflammatory mechanism (Fadok et al., 1998; Arabpour et al., 2021). As a result, we propose that the absence of polymorphonuclear or mononuclear inflammatory infiltrates could be associated with TGF-β activation in MFS. (Milewicz et al., 2021).

Neptune ER, Frischmeyer PA, Arking DE, Myers L, Bunton TE, Gayraud B, Ramirez F, Sakai LY, Dietz HC. Dysregulation of TGF-beta activation contributes to pathogenesis in Marfan syndrome. Nat Genet. 2003 Mar;33(3):407-11. doi: 10.1038/ng1116.

Milewicz DM, Braverman AC, De Backer J, Morris SA, Boileau C, Maumenee IH, Jondeau G, Evangelista A, Pyeritz RE. Marfan syndrome. Nat Rev Dis Primers. 2021 Sep 2;7(1):64. doi: 10.1038/s41572-021-00298-7.

Fadok VA, Bratton DL, Konowal A, Freed PW, Westcott JY, Henson PM. Macrophages that have ingested apoptotic cells in vitro inhibit proinflammatory cytokine production through autocrine/paracrine mechanisms involving TGF-β, PGE2, and PAF, J. Clin. Invest., 1998, vol. 101 (pg. 890-898).

Arabpour M, Saghazadeh A, Rezaei N. Anti-inflammatory and M2 macrophage polarization-promoting effect of mesenchymal stem cell-derived exosomes. Int Immunopharmacol. 2021 Aug;97:107823. doi: 10.1016/j.intimp.2021.107823.

**How is the blood pressure in these Marfan mice?**

Despite the strong correlation between renal injury and blood pressure, mean arterial pressure was not evaluated in this study due to the possibility that invasive probe placement (Drost CJ 2019) could negatively affect renal micro- and macrocirculatory hemodynamics. However, the blood flow and spectral curve of the abdominal aorta were evaluated by placing a periaortic probe around it right above the inferior infra-phrenic artery.

Data analysis showed a significant reduction in aortic flow in the MFS group compared to the wild-type group (Figure below - B), thus justifying the reduction in renal artery flow in the MFS group. We have previously reported the aorta blood flow reduction in mg∆^lpn^ female mice (de Souza et al 2019) and it was attributed to high-output cardiac failure.

Additionally, we noticed continuous peak systolic velocity in the WT group as well as constant monophasic flow patterns and no alterations in the patterns of the relaxation curves. As opposed to the WT group, the MFS group displayed monophasic flow with variable peak systolic velocities and a lower systolic peak. Moreover, it was followed by altered diastolic phase peaks (Figure below - A).

Drost CJ. This handbook is an educational service provided. Transonic Systems Inc.®.2019.

Supplementary figure 2. Aorta Blood Flow. A. Spectral curve analysis of aortic blood flow, in the WT group, presents a uniform monophasic flow pattern with similar peak systolic velocities (PSVs) (black arrows) and no alterations in relaxation curves (black arrowhead). In MFS- we observe a PSV reduced (blue arrow), and alteration in the diastole curve (blue arrowhead). B. Quantification of the aorta blood flow revealed a significantly decreased in the MFS group when compared to the WT group. (*** ρ<0.0001) The statistical analysis was performed by the Mann-Whitney test

**Are the angiotensin II blood levels altered? It would be greatly interesting to include some of this data in the paper**

We are grateful for your suggestion and comments on adding the angiotensin II (Ang II) measure for improvement in our study.

Animals' plasma was not stored, making it impossible to measure the plasma levels of angiotensin II suggested by the referee. Therefore, we used the Zen software to determine the intensity of angiotensin II expression in kidney tissue via immunohistochemistry.

The marker of Ang II in kidney tissue was predominantly identified in the convoluted tubules in WT. On the other hand, Ang II expression was significantly reduced in the MFS group as compared to the WT.

These findings were consistent with those of Fountain and Lappin (2022), who observed a reduction of Ang II by angiotensinogen-converting enzyme inhibitors and angiotensin receptors, leading to reduction in blood pressure, in blood flow, and in arterial tones. Based on these findings, the significant reduction of Ang II in the MFS group appears to explain the reduction of renal artery blood flow and velocity in microcirculation in mgΔ^lpn^ mice, as well as reductions of the systolic peak in the aortic spectral curve.

Fountain JH, Lappin SL. Physiology, Renin Angiotensin System. 2022 Jun 18. In: StatPearls [Internet]. Treasure Island (FL): StatPearls Publishing; 2022 Jan–. PMID: 29261862.

Figure 8. Immunohistochemistry for Angiotensin II in kidney tissue. A. Ang II marker in convoluted tubules in WT and MFS groups. Qualitatively observed the wide stronger marker in the WT group. However, in the MFS group, the marker of the Ang II showed positive in some convoluted tubules. B. Quantification of the intensity of the Immunohistochemistry of the Ang II showed a significant reduction in the MFS group when compared to the WT group. Positive marker in brown. (***) p <0.001. The statistical analysis was performed by the Mann-Whitney test (C, E, and G).

The last comments:

One point that is not well explained is **when authors affirm that fragmentation of elastic fibers in the aorta is related to an increase in collagen type I…(Discussion section). In fact,** **usually (not always) collagen expression is increased to compensate in some extent the loss of elasticity**

As recommended, we changed the affirmation to a suggestion concerning collagen deposition in kidney tissue.

Reviewer #2: This is an interesting study providing compelling evidence of alterations in kidney morphology in a mutant fibrillin-1 mouse model. The authors clearly show that glomerular morphology is affected, and that fibrillin and elastin fibers are damaged in the Fbn1 mutants, associated with an increased deposit of collagen fibers. Renal blood flow was significantly reduced in the Fbn1 mutants, associated with an increase in microvascular density. Nevertheless, kidney function was apparently not affected in the mutant mouse model.

While the morphological phenotype in the kidney of the Fbn1 mutant mice is clearly demonstrated, it is **surprising that no functional differences were shown**. The filtration function of the glomeruli does not appear to be decreased in the Fbn1 mutant mice, **since serum creatinine and urea are not different**. The authors **do not clearly address the possible reasons why this discrepancy exists between the striking morphological phenotype together with the strong reduction in renal blood flow and the lack of a functional phenotype in the kidney**. Besides the important filtration function, the kidney also plays a central **role in blood pressure regulation**. Renal blood flow is known to affect renin production. **Did the authors look at activity of the RAAS in the Fbn1 mutant mice?** At a minimum, **blood pressure measurements should be performed to investigate whether the reduction in renal blood flow leads to angiotensin-induced hypertension**. This could be an important mechanism **which might contribute to the cardiovascular pathophysiology in patients with Marfan syndrome (e.g. as described in DOI: 10.1007/s004670050327).** The authors make a similar statement at the end of the discussion, **but this is currently not supported by the data presented in the manuscript**.

A minor comment **relates to the choice of the mgΔlpn Fbn1 mutant model for this study. What was the rationale of the authors to use this specific model instead of more commonly used Fbn1 mutant mouse models? It would also be interesting to provide a more direct comparison to the previously published findings in the mgR/mgR model (DOI: 10.1007/s00428-004-1081-6), which seems quite similar to the phenotype described in this study**.

Other minor comments:

- In the results section on “Fibers of the Elastic Fiber System (FEFS) in the glomerulus”, the authors mention the existence of “an amorphous substance between the mesangial area and glomerular capillary”. **Can they elaborate on the potential type of substance observed here**?

- In the cluster analysis of the FEFS, the authors state that subgroup A consists of microfibrils, and subgroup C of elastic fibers. **What does subgroup B represent**? **And why were the MFS FEFS not divided in corresponding subgroups for the comparisons in Figure 2**?

- From the histological analysis shown in panel A of Figure 6, **the fragmentation of the elastic fibers is not very clear**. Perhaps a more detailed view can be shown, **or the elastic fibers can be marked more clearly**?

- **A limitation of the study is that only male mice were examined. Considering the known differences in the cardiovascular system, and especially related to the RAAS the authors should at least acknowledge this limitation**.

We are really pleased with the reviewer's assessment of our article *“This is an interesting study providing compelling evidence of alterations in kidney morphology in a mutant fibrillin-1 mouse model”.*

The Reviewer makes comments relating to explaining some morphologic/physiologic aspects.

While the morphological phenotype in the kidney of the Fbn1 mutant mice is clearly demonstrated, it is **surprising that no functional differences were shown**. The filtration function of the glomeruli does not appear to be decreased in the Fbn1 mutant mice, **since serum creatinine and urea are not different**. The authors **do not clearly address the possible reasons why this discrepancy exists between the striking morphological phenotype together with the strong reduction in renal blood flow and the lack of a functional phenotype in the kidney**.

Yes, we understand and acknowledge that it is important that you question the reason for the absence of renal dysfunction in the face of important morphological alterations, even those associated with reduced blood flow to the renal territory. A logical hypothesis could be developed regarding the ratio of the impaired and remaining integrity of the preserved renal functional units to the point where renal failure would occur. This theory was reinforced by the animals' apparently healthy clinical appearance and normal creatinine and urea levels, indicating preserved glomerular filtration in the absence of fibrosis in the renal tissue by histology, which is considered to be the final common pathway in the evolution of virtually all types of chronic kidney disease (Li et al., 2022). Certainly, the general findings allow us to hypothesize that the process of deterioration is ongoing but still compensated by the biological functional adaptive capacity.

Li, L., Fu, H. & Liu, Y. The fibrogenic niche in kidney fibrosis: components and mechanisms. Nat Rev Nephrol 18, 545–557 (2022). https://doi.org/10.1038/s41581-022-00590-z.

Renal blood flow is known to affect renin production**. Did the authors look at activity of the RAAS in the Fbn1 mutant mice?**

We have appreciated your suggestion to look at the activity of the RAAS in this study. In this study, we choose angiotensin II, because is considered one major compound of the RAAS (Fountain and Lappin 2022). In addition, the increase in Ang II has been associated with hypertension and the decrease in Ang II has been associated with a reduction in blood pressure, a reduction of blood flow, and a reduction in arterial tones (Prieto et al. 2021; Fountain and Lappin 2022).

In this study, we study the angiotensin II expression in kidney tissue via immunohistochemistry, and we measure the intensity of the marker by Zen software.

The marker of Ang II in kidney tissue was predominantly identified in the convoluted tubules in WT. On the other hand, Ang II expression was significantly reduced in the MFS group as compared to the WT.

These findings were consistent with those of Fountain and Lappin (2022), who observed a reduction of Ang II by angiotensinogen-converting enzyme inhibitors and angiotensin receptors, leading to a reduction in blood pressure, a reduction of blood flow, and a reduction in the arterial tones. Based on these findings, the significant reduction of Ang II in the MFS group appears to explain the reduction of renal artery blood flow and velocity in microcirculation in mgΔlpn mice, as well as reductions of the systolic peak in the aortic spectral curve.

Fountain JH, Lappin SL. Physiology, Renin Angiotensin System. 2022 Jun 18. In: StatPearls [Internet]. Treasure Island (FL): StatPearls Publishing; 2022 Jan–. PMID: 29261862.

Prieto MC, Gonzalez AA, Visniauskas B, Navar LG. The evolving complexity of the collecting duct renin-angiotensin system in hypertension. Nat Rev Nephrol. 2021 Jul;17(7):481-492. doi: 10.1038/s41581-021-00414-6.

Figure 8. Immunohistochemistry for Angiotensin II in kidney tissue. A. Ang II marker in convoluted tubules in WT and MFS groups. Qualitatively observed the wide stronger marker in the WT group. However, in the MFS group, the marker of the Ang II showed positive in some convoluted tubules. B. Quantification of the intensity of the Immunohistochemistry of the Ang II showed a significant reduction in the MFS group when compared to the WT group. Positive marker in brown. (***) p <0.001. The statistical analysis was performed by the Mann-Whitney test (C, E, and G).

At a minimum, **blood pressure measurements should be performed to investigate whether the reduction in renal blood flow leads to angiotensin-induced hypertension**. This could be an important mechanism that might contribute to the cardiovascular pathophysiology in patients with Marfan syndrome (e.g. as described in DOI: 10.1007/s004670050327). **The authors make a similar statement at the end of the discussion, but this is currently not supported by the data presented in the manuscript**.

Despite the strong correlation between renal injury and blood pressure, mean arterial pressure was not evaluated in this study due to the possibility that invasive probe placement (Drost CJ 2019) could negatively affect renal micro- and macrocirculatory hemodynamics. However, the blood flow and spectral curve of the abdominal aorta were evaluated by placing a periaortic probe around it right above the inferior infra-phrenic artery.

Data analysis showed a significant reduction in aortic flow in the MFS group compared to the wild-type group (Figure below - B), thus justifying the reduction in renal artery flow in the MFS group. We have previously reported the aorta blood flow reduction in mg∆^lpn^ female mice (de Souza et al 2019) and it was attributed to high-output cardiac failure.

Additionally, we noticed continuous peak systolic velocity in the WT group as well as constant monophasic flow patterns and no alterations in the patterns of the relaxation curves. As opposed to the WT group, the MFS group displayed monophasic flow with variable peak systolic velocities and a lower systolic peak. Moreover, it was followed by altered diastolic phase peaks (Figure below - A).

Drost CJ. This handbook is an educational service provided. Transonic Systems Inc.®.2019.

Supplementary figure 2. Aorta Blood Flow. A. Spectral curve analysis of aortic blood flow, in the WT group, presents a uniform monophasic flow pattern with similar peak systolic velocities (PSVs) (black arrows) and no alterations in relaxation curves (black arrowhead). In MFS- we observe a PSV reduced (blue arrow), and alteration in the diastole curve (blue arrowhead). B. Quantification of the aorta blood flow revealed a significantly decreased in the MFS group when compared to the WT group. (*** ρ<0.0001) The statistical analysis was performed by the Mann-Whitney test

A minor comment **relates to the choice of the mgΔlpn Fbn1 mutant model for this study. What was the rationale of the authors to use this specific model instead of more commonly used Fbn1 mutant mouse models? It would also be interesting to provide a more direct comparison to the previously published findings in the mgR/mgR model (DOI: 10.1007/s00428-004-1081-6), which seems quite similar to the phenotype described in this study**

We chose the mgΔ^lpn^ mice model because it has been generated by our group (Lima et al. 2010), and it is a dominant-negative model where heterozygotes present classical MFS phenotypes, including skeletal, ocular, and cardiovascular manifestations. In particular, the mgΔ^lpn^ presents aneurysm and aortic dissection similar to MFS patients (Lima et al. 2010; de Souza et al 2019; Souza et al., 2021a; Souza et a., 2021b). In contrast, the mgR/mgR mouse model has a hypomorphic *FBN1* mutation, and only homozygotes present skeletal and cardiovascular phenotypes.

We appreciate your suggestion to compare direct from both models mgR/mg R and mgΔ^lpn^. We added in the discussion sections the comparison of models.

**Line 303 to 308** “Although in the MFS group we observed alterations in structural components, no changes in renal functions were discovered. Hartner et al. [10] found in the mgR/mgR model, with lower expression of fibrillin-1, glomerular hypoplasia with decreased immunolocalization of fibrillin-1 in the renal parenchyma, as well as intact renal functionality. These findings support the hypothesis that alterations in fibrillin-1 are insufficient to cause a change in renal function, but they do cause an alteration in kidney structure”.

Franken, R., Heesterbeek, T. J., de Waard, V., Zwinderman, A. H., Pals, G., Mulder, B. J., & Groenink, M. (2014). Diagnosis and genetics of Marfan syndrome. Expert opinion on orphan drugs, 2(10), 1049-1062.

- In the results section on “Fibers of the Elastic Fiber System (FEFS) in the glomerulus”, the authors mention the existence of “an amorphous substance between the mesangial area and glomerular capillary”. **Can they elaborate on the potential type of substance observed here**?

The amorphous substances, which we indicated in TEM analysis could be possibly a basement membrane-like material (BM), due to localization. The presence of the amorphous material was referenced in subendothelial alterations in the glomerular basement membrane (Sbar et al., 1996 e Hsu e Churg 1979). We added in the manuscript the suggestion.

**Line 166 to 168**: … “Possibly the amorphous substance inside the glomerulus near the glomerular capillaries is a basement membrane-like material (BM). This sort of substance has already been identified in glomerular basement membrane subendothelial alterations. (Hsu e Churg 1979; Sbar et al., 1996)”

- In the cluster analysis of the FEFS, the authors state that subgroup A consists of microfibrils, and subgroup C of elastic fibers. **What does subgroup B represent**? **And why were the MFS FEFS not divided in corresponding subgroups for the comparisons in Figure 2**?

The cluster analysis automatically separated the elastic fiber measurements into three subgroups, of which, coincidentally, subgroup A has a similar thickness distribution to microfibrils and subgroup C to mature elastic fibers. Possibly, subgroup B can be considered a group of elastic fibers that are in the process of elastogenesis, or even elauninin fibers. However, morphologically we did not identify the classic aspects of elauninin fibers.

The cluster analysis was not performed in the MFS group. Because the disease can lead to a dubious structural feature of the elastic fibers without a precise understanding of the probable modifications. Possibly the changes in fibrillin-1, which in itself already alters the formation of elastic fibers, and possibly the proteolytic actions, such as the increased expression of MMP-9 (an enzyme that can degrade elastic fibers and interfere with elastogenesis).Both scenarios might lead to a dubious structural feature of the elastic fibers without a precise understanding of the probable modifications.

By automatically separating the WT group into three subgroups, we observed that there are different FEFS sizes, possibly indicating a complexity in the structural arrangements of the elastic fibers in the kidney. On the other hand, when comparing the three subgroups with the MFS group, we observed that the FEFS of the MFS group has a significant reduction when compared to subgroup C, indicating that the elastic fibers of the MFS group do not have the capacity to form mature elastic fibers. In addition, when we apply cluster analysis, we observed in the MFS group the subgroup predominance is B.

**aa**

**ba**

**ca**

- From the histological analysis shown in panel A of Figure 6, **the fragmentation of the elastic fibers is not very clear**. Perhaps a more detailed view can be shown, **or the elastic fibers can be marked more clearly**?

We appreciate the suggestion to change the microphotographs to contribute to the work. Attached we have added this photo to the image for a better representation of changes in the elastic fibers in the renal artery.
