# Supplementary material for: Extracellular matrix and vascular dynamics in the kidney of a murine model for Marfan syndrome
Source: PLoS One. 2023 May 9;18(5):e0285418. doi: 10.1371/journal.pone.0285418 (PMC10168582; doi:10.1371/journal.pone.0285418)
Supplement: Attachment — Submitted filename: Response to Reviewers.docx [file pone.0285418.s012.docx]

**Extracellular Matrix and Vascular Dynamics in the Kidney of a murine model for Marfan syndrome**

Rodrigo Barbosa de Souza^1^; Renan Barbosa Lemes^1^; Orestes Foresto-Neto^3^; Luara Lucena Cassiano^4^; Dieter P Reinhardt ^5^; Keith M. Meek^2^; Ivan Hong Jun Koh^6^; Philip N. Lewis*^2;^ Lygia V Pereira^1^.

Review Comments to the Author

Please use the space provided to explain your answers to the questions above. You may also include additional comments for the author, including concerns about dual publication, research ethics, or publication ethics. (Please upload your review as an attachment if it exceeds 20,000 characters)

We appreciate the reviewers' efforts in providing a new opportunity to improve the text and by providing constructive ideas. To accommodate all of their recommendations, we made significant adjustments to the text, which we address below. We hope that our paper is now of acceptable quality to be published as a PlosOne article.

Reviewer #1: All my comments have been satisfactorily addressed both in the response letter and the resubmitted.

We are very grateful for the pertinent comments that brought improvements to our work.

Reviewer #2: In the revised version the authors have provided some more experimental data to support their conclusions. The additional blood flow measurements and immunohistochemical staining of AngII are appreciated, but unfortunately do not entirely address the important question regarding the potential systemic effects of reduced renal blood flow.

On one hand, the measured reduction in aortic blood flow does indeed argue against a hypertensive phenotype in the mgΔ^lpn^ model. On the other hand, blood pressure is also dependent on vascular tone and it is possible that chronic hypertension has led to remodeling of the vascular wall, increasing stiffness and reducing luminal diameter, resulting in decreased flow despite elevated or maintained blood pressure. It is appreciated that invasive measurements of blood flow are not advised in this model, but perhaps non-invasive (tail-cuff) measurements are plausible in future studies, although they admittedly do lack the same level of precision as invasive measurements. It is recommended that the authors do not make definitive statements about systemic blood pressure without these measurements.

Another final concern is that the local kidney AngII levels which are suggested by the IHC stain shown in the revised manuscript do not necessarily reflect systemic RAAS activity. The intrarenal production of AngII can be considered as a separate system, independent of renin release from juxtaglomerular cells. Some evidence suggests that intrarenal AngII is predominantly regulated by angiotensinogen availability, since kidney renin activity is approximately 1000-fold higher than plasma renin activity. It is also not correct to link changes in kidney AngII to effects on aortic and renal blood flow, which are regulated by plasma AngII levels. Therefore, I recommend that the authors measure plasma renin concentration or plasma renin activity in this mouse model, particularly since reduced renal blood flow is the classical trigger for renin release from juxtaglomerular cells, which could then contribute to vessel damage by increased systemic AngII production.

We appreciate the reviewer's comments about adding the new data "In the revised version, the authors have provided some more experimental data to support their conclusions. The additional blood flow measurements and immunohistochemical staining of AngII are appreciated"

On one hand, the measured reduction in aortic blood flow does indeed argue against a hypertensive phenotype in the mgΔ^lpn^ model. On the other hand, blood pressure is also dependent on vascular tone and it is possible that chronic hypertension has led to remodeling of the vascular wall, increasing stiffness and reducing luminal diameter, resulting in decreased flow despite elevated or maintained blood pressure. It is appreciated that invasive measurements of blood flow are not advised in this model, but perhaps non-invasive (tail-cuff) measurements are plausible in future studies, although they admittedly do lack the same level of precision as invasive measurements. It is recommended that the authors do not make definitive statements about systemic blood pressure without these measurements.

We appreciate your interpretation of the relationship between the changed vascular tone and the reduced blood flow.

We were grateful for the proposal to assess systemic blood pressure using non-invasive (tail-cuff) readings; this is something we will do in future studies.

We'll revise the content to refrain from making categorical claims regarding systemic blood pressure.

Another final concern is that the local kidney AngII levels which are suggested by the IHC stain shown in the revised manuscript do not necessarily reflect systemic RAAS activity. The intrarenal production of AngII can be considered as a separate system, independent of renin release from juxtaglomerular cells. Some evidence suggests that intrarenal AngII is predominantly regulated by angiotensinogen availability, since kidney renin activity is approximately 1000-fold higher than plasma renin activity. It is also not correct to link changes in kidney AngII to effects on aortic and renal blood flow, which are regulated by plasma AngII levels. Therefore, I recommend that the authors measure plasma renin concentration or plasma renin activity in this mouse model, particularly since reduced renal blood flow is the classical trigger for renin release from juxtaglomerular cells, which could then contribute to vessel damage by increased systemic AngII production.

We appreciate your comments and suggestions about Ang II and renin plasma activity, respectively. However, the animals' plasma was not stored, making it impossible to measure any plasma components, as mentioned in the last review. Additionally, 6-month-old animals will be needed in order to analyze plasma renin, necessitating significantly longer study duration.

For this reason, we changed the text of the manuscript and made no statement about the relationship between systemic blood pressure and the activity of RAAS components. Besides, we would like to mention that this study was mainly focused on the descriptive aspect of the kidney in the mgΔ^lpn^ model, which may awaken as a finding for future studies, in addition to enabling new therapeutic targets and providing an insight into the pathophysiological mechanism of Marfan syndrome disease.

Line 362-370: “Furthermore, in the MFS group, a significant reduction of angiotensin II (AngII) was found in the renal convoluted tubule, which at first raised the possibility of a direct connection with the blood flow and arterial tone alterations. Alterations in intrarenal AngII have been described in some physiological conditions, such as in renal disfunction and hypertension. However, the precise contribution of intrarenal AngII in these conditions is poorly understood [63, 64, 65, 66]. As we did not measure the plasmatic components of the Renin-Angiotensin-Aldosterone system (RAAS) and blood pressure, we could not conjecture their relationship with the hemodynamic state. In future studies, these pathophysiological aspects need to be better exploited.”

63. Zhuo JL, Li XC. Novel roles of intracrine angiotensin II and signalling mechanisms in kidney cells. J Renin Angiotensin Aldosterone Syst. 2007 Mar;8(1):23-33. doi: 10.3317/jraas.2007.003.

64. Zhuo JL, Ferrao FM, Zheng Y, Li XC. New frontiers in the intrarenal Renin-Angiotensin system: a critical review of classical and new paradigms. Front Endocrinol (Lausanne). 2013 Nov 11;4:166. doi: 10.3389/fendo.2013.00166.

65. Li XC, Zhu D, Zheng X, Zhang J, Zhuo JL. Intratubular and intracellular renin-angiotensin system in the kidney: a unifying perspective in blood pressure control. Clin Sci (Lond). 2018 Jul 9;132(13):1383-1401. doi: 10.1042/CS20180121.

66. Navar LG, Prieto MC, Satou R, Kobori H. Intrarenal angiotensin II and its contribution to the genesis of chronic hypertension. Curr Opin Pharmacol. 2011 Apr;11(2):180-6. doi: 10.1016/j.coph.2011.01.009.
