# Supplementary material for: Extracellular matrix and vascular dynamics in the kidney of a murine model for Marfan syndrome
Source: PLoS One. 2023 May 9;18(5):e0285418. doi: 10.1371/journal.pone.0285418 (PMC10168582; doi:10.1371/journal.pone.0285418)
Supplement: Attachment — Submitted filename: Response to Reviewers.docx [file pone.0285418.s013.docx]

**Extracellular Matrix and Vascular Dynamics in the Kidney of a murine model for Marfan syndrome**

Rodrigo Barbosa de Souza^1^; Renan Barbosa Lemes^1^; Orestes Foresto-Neto^3^; Luara Lucena Cassiano^4^; Dieter P Reinhardt ^5^; Keith M. Meek^2^; Ivan Hong Jun Koh^6^; Philip N. Lewis*^2;^ Lygia V Pereira*^1^.

Review Comments to the Author

Please use the space provided to explain your answers to the questions above. You may also include additional comments for the author, including concerns about dual publication, research ethics, or publication ethics. (Please upload your review as an attachment if it exceeds 20,000 characters)

We appreciate the reviewers' efforts in providing a new opportunity to improve the text. To accommodate all of their recommendations, we made significant adjustments to the text and had it reviewed by a native English speaker. We hope that our paper is now of acceptable quality to be published as a PlosOne article.

Reviewer #2: I agree with the proposed changes to the discussion, weakening the statements regarding the connection between the intrarenal Ang II measurements and the hemodynamic state in the mice. I would suggest to also rephrase the rationale given on lines 289-292, where the link between Ang II and vascular tone / arterial pressure is highlighted. As the authors acknowledge in the revised discussion, intrarenal Ang II measurements will not address this issue, so this statement should also be amended not to confuse the reader.

We are grateful for the suggestions to improve the manuscript. We changed the text in lines 289 to 291.

Lines 289-291: Furthermore, due to significant reductions in blood flow in the MFS group, we analyzed angiotensin II (Ang II) in the kidney. Ang II is a major component of the renin-angiotensin-aldosterone system (RAAS), produced by convoluted tubules and juxtaglomerular cells [43, 44].

I have noticed that Supplementary Figure 2 (showing the aortic flow profile) is missing from the submission. There is a reference to it in the text and a legend is provided, but currently Fig S2 shows the creatinine and urea measurements, and Fig S3 shows the SDF video processing.

We apologize for the mistake; we included the necessary supplementary material.

Finally, It would also be good if the manuscript can be checked one last time by a native English speaker, as some sentences still contain some grammatical mistakes, particularly in the revised parts of the text.

We appreciate the reviewers’ comments. The Text was reviewed by a native English speaker.
